# Prognostic factors in the patients with T2N0M0 colorectal cancer

**DOI:** 10.1186/s12957-016-0826-4

**Published:** 2016-03-10

**Authors:** Bin Xu, Lin Yu, Li-Zhong Zhao, Dong-Wang Ma

**Affiliations:** Department of Colorectal Surgery, Tianjin Union Medical Center, Jie-yuan Road, Hong-qiao District, Tianjin 300191 China; Research Institute of Anal and Colorectal Disease of Tianjin City, Tianjin Union Medical Center, Jie-yuan Road, Hong-qiao District, Tianjin 300191 China

**Keywords:** Colorectal carcinoma, Prognosis, Lymphovascular permeation, Perineural invasion, Differentiation

## Abstract

**Background:**

The 5-year survival rate of the patients with stage I colorectal cancer is about 90 %; therefore, adjuvant therapy has not been recommended after radical resection; however, about 16–26 % of T2N0M0 patients will be dead at 5 years despite radical curative resection. It indicated that there is a defined group of patients who are at high risk for relapse or metastasis despite radical operation. This study aimed to find the patients with T2N0M0 colorectal cancer at high risk for relapse or metastasis.

**Methods:**

From January 1993 to December 2014, 812 patients with histologically confirmed stage T2N0M0 primary colorectal cancer treated by radical surgery with complete clinical follow-up data were eligible for this study. The medical records of all patients were collected and were retrospectively analyzed. Survival rates were calculated using Kaplan-Meier method, and survival cures were compared using the log-rank test. Cox proportional hazards model was used to analyze the significant factors defined in univariate test.

**Results:**

The 5-year and 10-year overall survival rates were 81.9 and 67.7 %, respectively. Male gender, old age, lymphovascular permeation, perineural invasion, and poor differentiation were associated with low cancer-specific survival rates in Kaplan-Meier analysis. Multivariate analyses revealed old age, lymphovascular permeation, perineural invasion, and poor differentiation as significant independent factors predicting worse prognosis (*P* < 0.05).

**Conclusions:**

Old age, lymphovascular permeation, perineural invasion, and poor differentiation are risk factors for the worse prognostic patients with T2N0M0 colorectal patients who would potential benefit from more aggressive therapy.

## Background

Colorectal cancer has been one of the most common cancers in the world, especially in developed countries [1–3]. The incidence and mortality of colorectal cancer has increased over the past 20 years, and it would increase consecutively if no effective action on colorectal cancer control. Colorectal cancer was the sixth most common cancer in China. It was estimated that there were 274,841 new cases diagnosed in 2010 (157,355 in males and 117,486 in females), with the crude incidence rate of 20.1/100,000, highest in males in urban areas. Age-standardized rates by China standard population of 2000 (ASRcn) and World standard population (Segi’s population, ASRwld) for incidence were 16.1/100,000 and 15.9/100,000, respectively. There were 132,110 cases estimated to have died from colorectal cancer in China in 2010 (76,646 men and 55,464 women) with the crude mortality rate of 10.1/100,000. The ASRcn and ASRwld for mortality were 7.55/100,000 and 7.44/100,000, respectively, higher in males and urban areas than in females and rural areas [4].

Approximately one tenth of colorectal cancer patients present with stage I disease. For these patients, the 5-year survival rate is about 90 %; therefore, adjuvant therapy has not been recommended after radical resection; however, about 16–26 % of T2N0M0 patients will be dead at 5 years despite radical curative resection [5, 6]. Furthermore, a nested reverse transcriptase-polymerase chain reaction was used to detect the circulating tumor cell by using CEA as tumor cell marker. The positive rate in patients with Dukes A was 38 %. It indicated that there is a defined group of patients who are at high risk for relapse despite radical operation [7].

To identify a subset of patients at high risk for recurrence and tumor-related mortality, we have evaluated treatment results and prognostic factors for patients with T2N0M0 colorectal cancer treated by radical resection.

## Methods

From January 1993 to December 2014, patients with histologically confirmed stage T2N0M0 primary colorectal cancer treated by radical surgery in Tianjin Union Medical Center were eligible for this study. The medical records of all patients were collected and were retrospectively analyzed. Because chemoradiotherapy may affect the number of lymph nodes and the metastatic pattern [8], patients with colorectal cancer undergoing neoadjuvant therapy were excluded. The study protocol was approved by the local ethics committee (Research ethics committee of Tianjin Union Medical Center).

The preoperative blood test included the level of CEA and CA19-9. All patients underwent a radical operation. The specimens were examined for tumor invasive depth, macroscopic type, tumor diameter, histological type, differentiation, number of involved lymph nodes, lymphovascular invasion, and perineural invasion. The specimens were staged according to the AJCC and UICC system (2010) after the final histopathological examination. The patients were followed at 3-month interval for 2 years, at 6-month interval for the next 3 years, thereafter yearly. Follow-up was history, physical examination, serum carcinoembryonic antigen and CA19-9 assay, chest X-ray, liver ultrasound, and abdominopelvic computed tomography, as possible. Surveillance colonoscopy was performed annually.

Statistical analysis was conducted using SPSS software (SPSS for windows version 17.0, Chicago, IL). Survival rates were calculated using Kaplan-Meier method, and survival cures were compared using the log-rank test. Cox proportional hazards model was used to analyze the significant factors defined in univariate test. Significant independent factors for overall survival were defined. Chi-square analysis and *t* test were used to comparing factors between different groups. A *P* value less than 0.05 was considered statistically significant.

## Results

### Patient characteristics and pathologic variables

The clinical characteristics and pathologic variables of the 812 patients with complete clinical follow-up data for analysis are summarized in Table 1. The median age was 60 years (range, 24–88 years). The operations underwent on all patients were with curative intent including right colectomy (4.7 %, 38/812), extended right colectomy (0.2 %, 2/812), left colectomy (1.7 %, 14/812), sigmoid colectomy (3.9 %, 36/812), total colectomy, abdominoperineal resection (51.1 %, 415/812), Dixon operation (36.3 %, 295/812), and Harrtmann (0.2 %, 2/812) operation (1.2 %, 16/812). All reported margins were negative for tumor involvement. The median tumor diameter was 30 mm (range, 5–70 mm). During the follow-up period of 130 months (range, 6–234 months), the 5- and 10-year overall survival rates were 81.9 and 67.7 %, respectively.

**Table 1:** Univariate analyses of factors for overall survival (OS)

	Number (%)	5-year OS (%)	10-year OS (%)	*χ* ^2^	*P*
Gender					
Male	435 (53.6)	77.0	63.9	6.379	0.012
Female	377 (46.4)	86.4	71.1		
Age (years)					
≤35	32 (4.0)	84.0	80.6	45.128	0.000
36–64	470 (57.9)	82.1	76.0		
≥65	310 (38.2)	72.2	49.6		
Depth of tumor invasion					
Superficial muscle	320 (39.4)	82.7	67.6	2.071	0.156
Deep muscle	492 (60.6)	80.3	65.7		
Lymphovascular permeation					
Positive	60 (7.4)	69.2	50.4	11.075	0.001
Negative	752 (92.6.)	83.5	69.5		
Perineural invasion					
Positive	31 (3.8)	68.2	54.6	7.769	0.005
Negative	781 (96.2.)	83.1	68.4		
Differentiation					
Well	207 (25.5)	86.7	73.7	15.509	0.000
Moderate	456 (56.2)	81.7	66.7		
Poor	135 (16.6)	71.1	48.5		
Mucinous	14 (1.7)	67.7	14.3		
Bowel obstruction					
Yes	90 (1.7)	81.7	68.9	1.06	0.304
No	722 (1.7)	83.5	70.0		
Location					
Colon	183 (11.1)	86.5	73.6	1.56	0.212
Rectum	629 (88.9)	80.3	65.6		
Preoperative CEA level(ng/ml)					
<5	299 (36.8)	82.0	66.8	0.583	0.747
≥5	513 (63.2)	81.2	67.7		
Preoperative CA19-9 level(ng/ml)					
<37	643 (79.2)	81.6	67.8	1.92	0.383
≥37	169 (20.8)	82.1	63.3		

### Univariate analyses

Univariate analyses of factors affecting survival are list in Table 1. Using Kaplan-Meier analysis for overall survival rate, we found that male gender, old age, lymphovascular permeation, perineural invasion, and poor differentiation were associated with low cancer-specific survival rates (*P* < 0.05). However, no statistically significant difference regarding depth of tumor invasion, bowel obstruction, colorectal tumor locations, preoperative serum CEA level, and CA19-9 level were observed (*P* > 0.05).

### Multivariate analyses

A Cox multiple regression model was used to assess the influence of all significant covariates on survival. In the proportional hazards model analysis, old age, lymphovascular permeation, perineural invasion, and poor differentiation were significant independent factors predicting worse prognosis (*P* < 0.05) (Table 2).

**Table 2:** Multivariate analyses of risk factors for overall survival (OS)

Variables	*β*	SE	Wald	*P*	Exp(*β*)
Differentiation	0.53	0.122	18.889	0.000	1.689
Perineural invasion	1.061	0.308	11.871	0.01	2.888
Lymphovascular permeation	0.932	0.244	14.559	0.02	2.541
Age	0.831	0.143	34.026	0.000	2.294

## Discussion

Colorectal cancer is one of the most commonly diagnosed cancers in the world. Over the last decade, total morbidity and mortality rates of colorectal cancer are increasing steadily in Asia countries, especially in China, colorectal cancer has become a fatal disease. T2N0M0 colorectal cancers are usually considered less advanced and are usually associated with favorable prognosis. However, once the tumor invades through the muscularis mucosa, metastasis to distant organs can occur. And moreover, in this study, the 10-year overall survival rate was 67.7 % despite radical resection. The Swedish Rectal Cancer Trial showed a significant reduction in LR in stage I patients with preoperative pelvic radiation, from 12 to 4 % [9].Indiscriminate use is not recommended in the early-stage patients owing to the over treatment of the majority, although adjuvant therapy may clearly benefit some of this population. Our study’s primary objective was to find a high-risk group of T2N0M0 patients using routine clinical and pathologic factors so that the salutary effects of adjuvant therapy might be extended to the patients.

Tumor differentiation is one of common pathologic variables. In our study, patients with poorly differentiated tumors also fared worse. Poorly differentiated tumors have a worse prognosis compared with better differentiated ones [10].Poorly differentiated colorectal cancers has been shown to correlate with bowel penetration, lymph node involvement, and vascular invasion, indicating that it is a risk factor for dissemination of colorectal cancer [11]. Other clinical studies have demonstrated that patients with poorly differentiated cancers have worse prognosis [12, 13].

Our results indicate that lymphovascular permeation has been linked with poor prognosis. Lymphovascular invasion can occur intramurally within colorectal wall itself or in the surrounding tissue. Although arterial invasion occurs, most series define and describe vascular invasion based on venous invasion. Venous invasion in colon cancer occurs in 42 % of patients and increases with increasing grade and stage [14]. Iinuma et al. [15] found venous invasions significantly correlated with the presence of isolated tumor cells in blood samples. Furthermore, another study reported that detecting circulating tumor cells in the peripheral blood was as a useful tool for determining the patients at high risk for recurrence [16]. These studies support the hypothesis that venous invasion is an essential step in the process of hematogenous metastasis. Patients with blood vessel invasion had a 74 % survival compared to those without it at 85 %. In those patients with both intramural and extramural vascular invasion, the prognosis was even worse at 32 %. Lymphatic invasion is the most common mechanism leading to metastatic disease. Lymphatic exist within the colorectal wall and lymphatic invasion correlates with the depth of penetration of colon cancers. T2 tumors have a risk of lymph node involvement up to 25 %. The presence of lymphovascular permeation was the only independent factor associated with a higher incidence of lymph node metastasis on multivariate analysis (odds ratio 1.48, 95 % CI 1.44–13.47, *P* = 0.009) [17]. The lymphatic drainage goes along the venous drainage of the colon and rectum, ultimately coursing through the portal vein and into the liver. Metastatic liver disease is felt to occur typically due to lymphatic spread. Furthermore, vascular invasion and vascular endothelial growth factor overexpression were all considerably correlated to the higher postoperative relapse rate and poorer overall survival rates in colorectal cancer patients after curative resection [18, 19].

Perineural invasion (PNI) is a pathologic process characterized by tumor invasion of nervous structures and spread along nerve sheaths. The pathogenesis of PNI likely involves complex signaling between tumor cells, the nerves, and stromal cells, but this area of research is still largely in its infancy [20–23]. PNI is known as a marker for a more aggressive tumor phenotype and poor prognosis in several malignancies, most notably head and neck and prostate cancers [24–28]. Perineural invasion also increases with increasing grade and stage of the tumor. It occurs in 14–32 % of colorectal cancers and can extend to as far as 10 cm from the primary tumor. Numerous studies have confirmed poorer prognosis when perineural invasion is noted.

Liebig et al. [23] found that disease-free and overall survival rates were significantly affected by PNI status independent of tumor stage in colorectal patients. Among node-negative patients, the 5-year disease-free survival for PNI-negative patients was almost three-fold greater than for PNI positive patients (82 vs. 29 %, respectively). In fact, node-negative patients with PNI had a significantly worse disease-free survival rate than node-positive patients (29 vs. 56 %). Similar results were seen for overall survival, where the 5-year overall survival for node-negative patients with PNI positive tumors was 43 % compared with 87 % for patients with PNI negative tumors. Similarly, node-negative but PNI-positive patients had a significantly lower 5-year overall survival rate compared with node-positive patients (43 vs. 67 %). A meta-analysis indicated that PNI was a poor prognostic factor in CRC patients, and the postoperative survival of stage II CRC patients with PNI(+) was probably more similar to that of stage III patients [29].

Younger age is a positive prognostic factor for patients with colorectal cancer [30–32]. In this study, younger patients were found to have better overall survival both in the univariate and multivariate analysis. Previous studies found that younger patients had worse prognosis [33], because younger patients were found to have more locally advanced, higher stage, and less favorable histologic subtypes tumors than older patients at the time of diagnosis [34–38]. It is not clear why younger patients tend to present with more advanced disease. Perhaps, because of their young age, physicians are less likely to suspect malignant disease than in older patients, thus leading to a delay in appropriate investigations and diagnosis. Another potential explanation is that younger patients would generally not to be included in CRC screening initiatives and would be less likely to have early cancers diagnosed through these screening initiatives. Furthermore, younger patients were treated more aggressively. This may partly explain their improved prognosis. In recent studies, when controlling for tumor stage, patient, and treatment factors, young patients had a superior overall survival [39–41].

The relationship between survival after surgical resection of colorectal carcinoma and perioperative blood transfusion was studied in many researches. Perioperative blood transfusion could impact immune function, increased postoperative mortality, local recurrence rate, and distant metastasis rate in colorectal cancer patients [42]. The combination of perioperative blood transfusion and subsequent development of postoperative infectious complications may be associated with a poor prognosis [43]. In our study, there were only six blood transfused patients, so we could not analyze further.

## Conclusions

In conclusion, poor differentiation, old age, lymphovascular permeation, and perineural invasion are associated with high risk for recurrence or metastasis in patients with T2N0M0 colorectal caner. Through the evaluation of routine clinical and pathologic data for patients treated with curative surgery, this study has defined a subpopulation of patients with T2N0M0 colorectal cancer that are at increased risk of mortality. These findings could identify patients with T2N0M0 colorectal cancer who might benefit from more aggressive therapy.
